# Graphene Nanosheets to Improve Physico-Mechanical Properties of Bioactive Calcium Silicate Cements

**DOI:** 10.3390/ma10060606

**Published:** 2017-05-31

**Authors:** Nileshkumar Dubey, Sneha Sundar Rajan, Yuri Dal Bello, Kyung-San Min, Vinicius Rosa

**Affiliations:** 1 Faculty of Dentistry, National University of Singapore, Singapore 119083, Singapore; a0121941@u.nus.edu (N.D.); sneha.xanotor@gmail.com (S.S.R.); 2 Faculty of Dentistry, University of Passo Fundo, Passo Fundo 99052-900, Brazil; yuri@upf.br; 3 School of Dentistry and Institute of Oral Bioscience, Chonbuk National University, Jeonju-si 54896, Korea; endomin@gmail.com; 4 Centre for Advanced 2D Materials and Graphene Research Centre, National University of Singapore, Singapore 117546, Singapore

**Keywords:** graphene, Biodentine, dental pulp stem cells, push-out bond strength, mineral trioxide aggregate

## Abstract

Bioactive calcium silicate cements are widely used to induce mineralization, to cement prosthetic parts, in the management of tooth perforations, and other areas. Nonetheless, they can present clinical disadvantages, such as long setting time and modest physico-mechanical properties. The objective of this work was to evaluate the potential of graphene nanosheets (GNS) to improve two bioactive cements. GNS were obtained via reduction of graphite oxide. GNS were mixed (1, 3, 5, and 7 wt %) with Biodentine (BIO) and Endocem Zr (ECZ), and the effects on setting time, hardness, push-out strength, pH profile, cell proliferation, and mineralization were evaluated. Statistics were performed with two-way ANOVA and Tukey test (α = 0.05). GNS has not interfered in the composition of the set cements as confirmed by Raman, FT-IR and XRD. GNS (1 and 3 wt %) shortened the setting time, increased hardness of both materials but decreased significantly the push-out strength of ECZ. pH was not affected but 1 wt % and 7 wt % to ECZ and 5 wt % to BIO increased the mineralization compared to the controls. In summary, GNS may be an alternative to improve the physico-mechanical properties and bioactivity of cements. Nonetheless, the use of GNS may not be advised for all materials when effective bonding is a concern.

## 1. Introduction

Bioactive cements are widely used for the management of perforations, retrograde root filling, pulp capping, cementation of prosthetic parts, and others [1,2,3]. Biodentine (BIO) and Endocem-Zr (ECZ) are different from the usual bioactive calcium-silicate “Portland cement” materials as it contains zirconia oxide instead of bismuth oxide as the radiopacifying agent. It is the safest bioactive cements that exhibits the least discoloration and calcification of the tooth and pulp chamber, respectively [4,5]. These bioactive cements can induce higher expression of several genes (e.g., bone sialoprotein, dentin sialophosphoprotein, alkaline phosphatase, osteocalcin) and phosphatase activity, hence, stimulating biomineralization [6,7,8,9]. Nonetheless, these cements may present some clinical disadvantages, such as long setting time and modest physico-mechanical properties [9,10]. Thus, the development of materials and strategies that can improve these aspects without compromising the bioactivity of cements is of high interest.

The addition of nanomaterials into biocements can improve their physical and chemical properties. For example, nanosilica promotes early precipitation and shortens the induction period of Portland cement (42.5 grade) decreasing the setting time and increasing the compressive strength [11]. The addition of 15 vol % of zirconia and 30 vol % of alumina to hydroxyapatite increased the flexural strength and fracture toughness by almost three times compared to pure hydroxyapatite [12]. Similar improvement can be observed with the addition of single-walled carbon nanotubes (CNTs) which accelerates the hydration of cements at an early age [13]. Furthermore, the addition of multi-walled CNTs can improve both the compressive strength and modulus of rupture of cementitious materials [14,15,16].

Graphene is a novel nanomaterial that has large surface area, high elastic modulus, flexural strength, and others [17]. Pristine graphene films can increase osteogenic differentiation of stem cells [18], while graphene oxide (GO) has been recently shown to increase the expression of osteogenic and odontogenic-related genes when used as a substrate to culture dental pulp stem cells (DPSCs) [19]. Additionally, these materials have been previously shown to improve physical and mechanical properties of cementitious products. For example, GO can increase the compressive strength of ordinary Portland cement by 33% and the flexural strength by 59% [20]. Similarly, the addition of GO to Portland cement paste can increase the compressive and tensile strength by 46% and 53%, respectively [21]. In fact, the improvements in mechanical properties of Portland cements by GO is concentration-dependent. The addition of GO can increase both the compressive and flexural strengths by 72% and 63% after seven days from mixing [22]. The presence of 1 wt % graphene nanosheets (GNS) in HAp-based composites have shown to improve the Vickers hardness by 30% compared to pure hydroxyapatite [23]. Also, the incorporation of 1 wt % GNS in calcium silicate ceramic composite have increase the fracture toughness by 130% [24]. Furthermore, 1 wt % GNS addition in bioceramics have promoted cell viability and cell proliferation and improved apatite mineralization compared to bioceramic alone [23,24]. Despite the improvements provided by different forms of graphene in cementitious materials, the potential of graphene-based materials to improve properties of bioactive cements remains largely unknown. 

The objective of this work was to evaluate the effects of graphene nanosheets on the bioactivity, physicomechanical and chemical properties of two bioactive cements. The hypothesis tested was that GNS can increase all the properties studied in a dose-dependent manner without compromising the materials’ bioactivity.

## 2. Results

**Figure 1:**
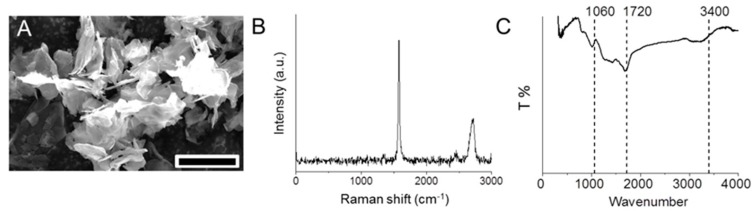
GNS characterization via SEM (**A**), scale bar = 10 µm; Raman (**B**); and FT-IR (**C**) spectroscopy.

GNS with several micrometers in lateral size was successfully produced through the chemical reduction of GO using hydrazine vapor (Figure 1A). Raman spectroscopy (Figure 1B) showed two high intensity peaks at 1583 and 2700 cm^−1^ explicitly for G and 2D bands. The G band depicts the stretching of the C–C bond in graphitic materials and 2D band is common to all sp^2^ carbon systems and is used to determine the number of graphene layers. The peak observed at 1060 cm^−1^ in the Fourier transform infrared spectroscopy (FT-IR) spectrum (Figure 1C) indicates structural vibrations from unoxidized graphitic domain and remaining carbonyl groups after the reduction process. The C=O at 1720 cm^−1^ is due to the peeling of graphitic structures through the insertion of oxygen between the graphene sheets during oxidation process [25]. The spectra show a peak at 3400 cm^−1^ corresponding to the stretching and bending vibration of O–H groups of water molecules adsorbed on graphene oxide. All of the characteristic absorption bands of oxygen-containing groups (O–H, C=O, and C–O) are substantially weakened and almost eliminated (Figure 1C). This confirms the reduction of graphene oxide to graphene nanosheets. 

**Figure 2:**
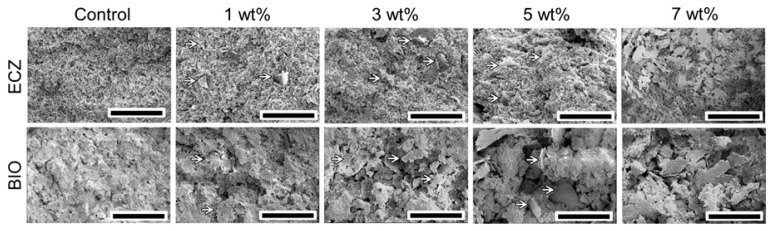
SEM of fracture surface of specimens showed that GNS (arrows) was evenly dispersed in the cements (scale bar = 50 µm).

**Figure 3:**
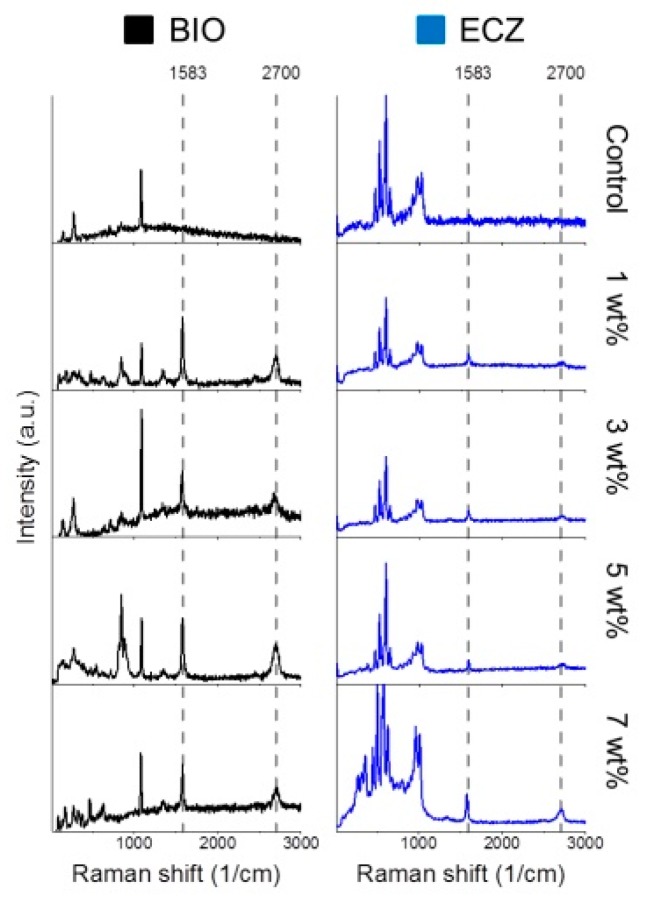
Raman spectroscopy shows the presence of peaks at 1583 and 2700 cm^−1^ for all of the experimental groups.

The microstructures of both biocements with different proportions of GNS are shown in Figure 2. Both biocements showed the presence of peaks at ~830 and 850 cm^−1^ corresponding to tri- and di-calcium silicate. The mixing procedures and setting reaction did not change GNS structure as the Raman spectroscopy confirmed the presence of peaks at 1583 and 2700 cm^−1^ for all the experimental groups (Figure 3). It is possible to observe that GNS kept its sheet-like shape and was evenly dispersed within the cements matrices after mixing (Figure 2). 

**Figure 4:**
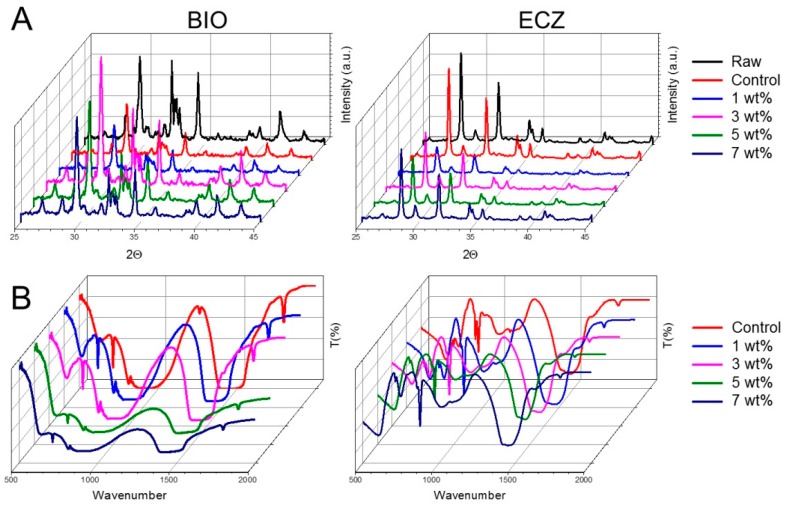
XRD (**A**) and FT-IR (**B**) for all conditions tested (Raw = unmodified powder before mixing).

The crystalline phases of BIO and ECZ with different GNS contents were investigated by X-ray powder diffraction (XRD) (Figure 4A). Both materials exhibited peaks for calcium carbonate (CaCO_3_) at 29.35°. Zirconium oxide (ZrO_2_) peaks were observed at 8.17° and 31.47° in both materials but more intensively in ECZ. The ZrO_2_ acts as a radio pacifier in substitution for bismuth oxide present in mineral trioxide aggregate (MTA) [26]. Though the intensities of the peaks varied with the addition of GNS, there was no extinction or shift of any peak compared to the unmodified materials (Control) suggesting that the hydration was not negatively affected by the presence of GNS.

The FTIR analysis was performed to determine the spectral changes between both BIO and ECZ with the incorporated GNS (Figure 4B). The cements showed the presence of calcium hydroxide (Ca(OH)_2_) with peaks between at 3640 cm^−1^. Furthermore, BIO and ECZ showed the presence of calcium carbonate peaks around 700–850 cm^−1^ and 1400–1480 cm^−1^ occurring because of the stretching and bending of –CO_3_^2−^ [27]. The presence of tricalcium silicate was observed in BIO with a peak around 850–874 cm^−1^ occurring due to stretching of –SiO_4_^4−^. Additional –OH group is also seen around 1640 cm^−1^ in both materials depicting the H–O–H bending vibrations of H_2_O molecules [28]. Moreover, the characteristic peaks of GNS was recognized as the symmetric stretching of CH2 at 2850 cm^−1^ and asymmetric stretching of CH2 at 2920 cm^−1^ at the edges/defects. 

**Figure 5:**
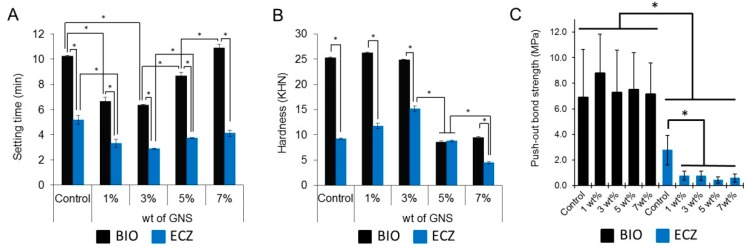
Setting time (**A**); hardness (**B**); and push-out bond strength (**C**) of the groups tested (* represents statistical significance between groups, *p* < 0.05). The fracture modes obtained in the push-out test are available in Figure S1.

Setting time, hardness, and push-out bond strength are shown in Figure 5. BIO presented longer setting time (A), higher hardness (B), and push-out bond strength (C) comparing to ECZ for all the conditions tested (*p* < 0.05). The addition of GNS to ECZ resulted in significant decrease of push-out bond strength for all the weight percentages tested (C). The fracture patterns obtained from the push-out test are presented in the Figure S1.

**Figure 6:**
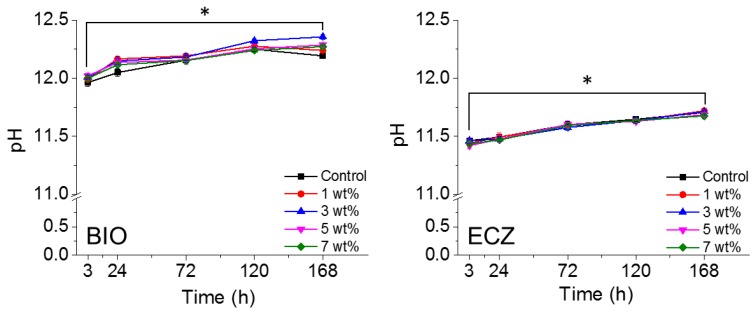
pH variation of both materials with different wt % of GNS. The addition of GNS did not change the alkaline potential of the materials tested (* represents statistical significance between groups, *p* < 0.05).

**Figure 7:**
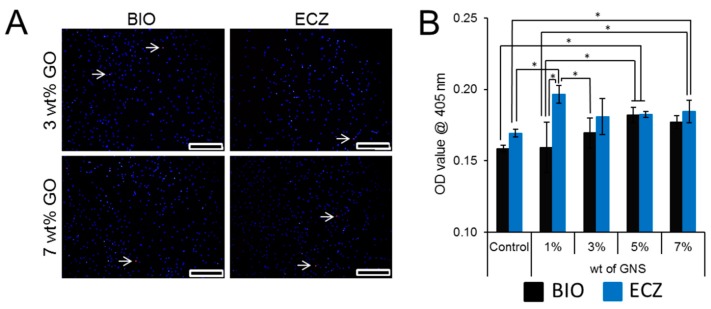
Cell viability was not compromised by the addition of 3 and 7 wt % of GNS after five days. The arrow indicates the dead cell in culture (**A**); bioactivity was assessed by Alizarin red S staining. There was no statistical difference for BIO and ECZ for the same condition except for 1 wt % of GNS (**B**). Scale bar = 20 µm, the full set of data for three and five days for cell viability is available in Figure S2.

The pH variation of both materials with different weight percentages of GNS is shown in Figure 6. The addition of GNS did not change the alkaline potential of the materials tested (* represents statistical significance between groups, *p* < 0.05). Fluorescence micrographs (Figure 7A) showed that DPSCs were successfully attached and did not exhibit any obvious effects on cell proliferation when treated with GNS-bioactive cement extract. Bioactivity was assessed by Alizarin red S staining. There was no statistical difference for the absorbance values observed for BIO and ECZ for the same condition except for 1 wt % of GNS (Figure 7B). The full set of data for cell viability is available in Figure S2.

## 3. Discussion

Bioactive cements have been used as a root end filling material and prospective studies have reported a failure of 9.8–16% at one year [29,30]. The incorporation of graphene-based material has been proposed for many materials, such as Portland cements and bioceramics, to improve mechanical properties and reduce setting time [20,21,23,24]. In this regard, the addition of GNS to bioactive cements should improve the physico-mechanical properties, proliferation, and mineralization potential to extend their clinical use.

The addition of GNS did not change the composition of crystalline phases of both bioactive cements tested (Figure 4B). A similar trend has been observed for Portland cements where the addition of GO did not result in difference in the positions of the XRD peaks [22]. The FTIR analysis (Figure 4B) showed that the spectrum of the mixed cement reinforced with different proportions of GNS exhibit bands comparable with those of the unmodified cements (Control). These confirm that the GNS do not chemically react with the cements during their setting reaction.

Regardless the addition of GNS, ECZ presented significant shorter setting time and lower hardness as compared to BIO (Figure 5A,B). The faster setting reaction of ECZ can be attributed to the presence of small particles of pozzolan cement which increases the surface contact of the particles with the mixing liquid and provides rapid setting and ease of handling [31]. It is noteworthy that BIO also presented relatively short setting time compared to other MTA-based materials that can take twice as much time to complete the setting reaction [9,31]. This can be partially explained by the presence of calcium carbonate in BIO which acts as a nucleation site for calcium silicate hydrate (C–S–H), thereby reducing the duration of the induction period [32]. The addition of 3 wt % of GNS to the mix resulted in significantly lower setting time for both materials compared to the control. It is feasible that carbon-based materials influence both the initial C_3_A and the C_3_S hydration products by accelerating the rate of hydration processes particularly at early stages since they act as a matrix for the development of C–S–H and calcium hydroxide [22,33,34]. The longer setting time observed for 5 and 7 wt % compared to 3 wt % may be related to the fact that, at high concentrations, carbon-based materials can agglomerate around cement grains allowing only partial hydration [35]. In this case, the hydrated product can present weak bonds which correlates to lower hardness values observed for 5 and 7 wt % (Figure 5B). The lower hardness observed for ECZ can also be related to the presence of ZrO_2_ (Figure 4A). It has been shown that when zirconia is used as a reinforcing agent in concentrations as high as 8 wt %, it can disrupt the hydroxyapatite microstructure reducing significantly the hardness of hydroxyapatite-based nanocomposites [36]. 

The effective bond of materials to dentin is crucial for the success of endodontic procedures. In general, BIO presented higher push-out bond strength as compared to ECZ regardless of the presence of GNS (Figure 5C). The presence of GNS decreased significantly the push-out strength of ECZ. Once there is no chemical reaction between GNS and ECZ (Figure 4A,B) it is possible that the GNS act as impurities within the cementitious matrix or prevent effective contact between ECZ and dentin resulting in lower push-out strength. High push-out bond strength has been previously reported for BIO compared to other root perforation repair materials such as Intermediate Restorative Material (IRM), MTA and others [37,38]. Interestingly, the GNS did not compromise the push-out strength for BIO. This can be related to the small particle size and uniform components of BIO that promote a good interlocking of material with dentine microstructure [38]. In addition, it may be related to the possible higher dissolution of Ca and Si ions in BIO that are exchanged with dentin resulting in the formation of tag-like structures in the material/dentin interface [39].

There was a significant increase in the pH of both biocements from 3 to 168 h for all of the conditions tested (Figure 6). In addition, BIO presented consistently higher pH for all the conditions and time points tested compared to ECZ. The increase of pH is related to the hydration of tricalcium silicate that results in the formation of calcium silicate hydrate gel and calcium hydroxide. The latter provides an alkaline pH in the surroundings upon Ca^2+^ dissolution [9,40]. The addition of GNS does not change the alkaline profile of the materials tested. This is of high importance for clinicians as the high pH can be associated with the antibacterial potential of these cements [41].

The effects of GNS in dental pulp stem cells proliferation was assessed using an indirect-direct method where the materials are soaked in basal culture medium and the extracts used to treat cells for a given period of time [42]. The addition of GNS did not increase the cytotoxicity of both materials after five days as few cells are stained with propidium iodide (white arrows in Figure 7A). Both BIO and ECZ can promote mineralization in DPSC as the absorbance obtained were statistically higher than the one obtained with basal medium (0.07 ± 0.01) although lower than with the use of osteogenic medium (0.40 ± 0.01). Although BIO presented higher pH than ECZ (Figure 6), the first failed to induce higher degrees of mineralization (Figure 7B). Although alkalinity plays an important in the mineral production, the differentiation of stem cells towards mineral-producing phenotype is also affected by the amount of calcium ions present in the microenvironment [9]. Surprisingly the addition of 1 and 7 wt % of GNS in ECZ was capable of increasing the mineralization of DPSC as compared to the control. For BIO, only the addition of 5 wt % was capable of exerting the similar response. Further studies are necessary to evaluate if these concentrations can increase calcium dissociation from these biocements. 

## 4. Materials and Methods

### 4.1. Sample Preparation

Multilayer graphene nanosheets (GNS) were synthesized by the reduction of GO as previously described [43]. GO was prepared via the modified Hummer’s method [19]. Briefly, 5 g of graphite (Lanka Graphite Ltd., Melbourne, Australia) were mixed with 2.5 g of NaNO_3_ in a solution of 12 mL H_3_PO_4_ and 108 mL H_2_SO_4_ in ice bath for 10 min. Following, 15 g of KMnO_4_ was gradually added and the suspension was stirred on ice bath for 2 h followed by additional 60 min at 40 °C. Subsequently, the temperature of the mixture was increased to 98 °C and kept for 60 min while deionized water was added to a final volume of 400 mL. After 5 min, 15 mL of H_2_O_2_ was added and the reaction product was centrifuged and washed with 5% HCl solution in deionized water. Finally, 0.1 g of graphite oxide was dispersed in 100 mL of deionized water via ultrasonication and hydrazine was added to promote the reduction of GNS at 80 °C. After, two bioactive cements namely BIO (Biodentine, Septodont, Saint-Maur-des-Fossés, France) and ECZ (EndocemZr, Maruchi, Gangwon-do, Korea) were combined with different proportions of GNS in powder form (1, 3, 5 and 7 wt %). BIO and ECZ without GNS were used as controls. For all assays, the powders were mixed per manufacturer instructions and transferred to molds. All of the mixtures were allowed to set for 24 h (37 °C, 95% humidity) prior testing (except for setting time, pH and push-out testing).

Sample characterization: Raman spectroscopy, Fourier transform infrared spectroscopy (FTIR), scanning electron microscopy (SEM), and X-ray diffraction (XRD)

Raman spectra were obtained using a CRM 200 (Witec, Ulm, Germany) connected to a microscope with 100× magnification. The spectra were obtained with an excitation laser source of 532 nm laser with a power of 0.1 mW for 10 s.

For SEM, disks were fractured and the cross-sections observed under SEM (Quanta 650 FEGSEM, FEI, Hillsboro, OR, USA). 

Crystalline phase analysis of the raw powder and set materials were obtained using an X-ray diffractometer (D8 Advance Powder X-ray Diffractometer, Bruker AXS, Karlsruhe, Germany) with a Ni filter and CuKa radiation (λ = 1.5425 Å) at 40 kV and 40 mA (scan range: 10–80°, scanning rate of 0.02°/s). Crystalline formations were identified using JCPDS standard data file. 

Infra-red spectra were obtained with FT-IR/NIR (Frontier, Perkin Elmer, Waltham, MA, USA) using FT-IR grade potassium bromide (KBr, Sigma-Aldrich, St. Louis, MO, USA) sample preparation technique. Samples were mixed with KBr and formed into pellets for analysis (five spectra for each specimen, spectral resolution of 2 cm^−1^, 100 scans per spectrum). 

### 4.2. Setting Time, Hardness, Push-Out Bond Strength, and pH

For setting time (*n* = 3), the materials were inserted into molds immediately after mixing and final setting time assessed every 60 s with a Gillmore needle (Gilson Company Inc, Delaware County, OH, USA) (453.6 g) according to ASTM C266-13 standard test method [44].

For Knoop hardness (HK), five indentations (100 g, 10 s) were made per specimen using Knoop diamond indenter (FM-100, Futuretech, Kawasaki, Japan) and HK calculated according to Equation (1) where *C_p_* is the correction factor related to the shape of the indenter (0.070279), *P* is the test load (kgf), and *L* is the length of the longer diagonal (µm):

(1) $$HK=\frac{P}{C_{p}L^{2}}$$

For pH variation, one disk (5 mm diameter × 1 mm thickness) of each group was placed individually in a tube containing 10 mL of ultrapure water (MilliQ, Millipore, Billerica, MA, USA) and stored at 37 °C. The pH of the solution was measured (Orion Star™ A211 pH Benchtop Meter, Thermo Scientific, Waltham, MA, USA) up to 168 h of incubation (*n* = 3). 

For push-out bond strength, the access to the pulp chamber of the premolars were prepared using a round diamond bur (#1012, KG Sorensen, São Paulo, Brazil) under water irrigation. The working length was determined with a K-file#10 (Maillefer Dentsply, Ballaigues, Switzerland) and root canals were instrumented up to #30 under irrigation with saline solution. Finally, the first 8 mm of the root canal was enlarged to a final diameter of 1.3 mm (Endo Niti Files Largo Peeso Reamer #4, Maillefer Dentsply, Ballaigues, Switzerland). Teeth were mounted in epoxy resin and sectioned horizontally (LabCut 1010 Low Speed Diamond Saw, Extec Corp, Enfield, CT, USA). The crown was removed and the root sliced into disks (2.0 ± 0.1 mm thick). The first cervical disk was rejected and the remaining used for push-out bond strength test (*n* = 10). The disks were filled with the cements and a lentulo spiral (#25/1, Maillefer Dentsply, Ballaigues, Switzerland) was used to release air bubbles. The teeth were wrapped in wet gauze and specimens stored for seven days in an incubator at 37 °C.

The dentin slices were tested for push-out bond strength (*n* = 10) as previously described by Valandro et al. [45]. The test was performed in a universal testing machine (EMIC DL2000, Paraná, Brazil) at 0.5 mm/min. The bond strength (*σ_PO_*, in MPa) was calculated according to Equation (2) where *F* is the load for specimen rupture (in N) and *A* is the bonded area (mm^2^):

(2) $$\sigma_{PO}=\frac{F}{A}$$

The bonded area (*A*) was determined using Equations (2) and (3), where *g* is the slant height, *R*1 is the smaller base radius, *R*2 is the larger base radius and h is the section height (all in mm). *R*1 and *R*2 were obtained under optical microscope (Z-TXE, Shangrao, China):

(3) A=πg(R1+R2)

(4) $$g={\left[h^{2}+{\left(R2-R1\right)}^{2}\right]}^{1/2}$$

### 4.3. Dental Pulp Stem Cells Culture, Cell Proliferation, and Bioactivity

The use of human monoradicular premolars and dental pulp stem cells (DPSC) for research was approved by the Institutional Review Board/NUS (Approval Number: NUS 2094). The DPSC (Allcells, Alameda, CA, USA) between passage 3 and 6 were used to assess the cell proliferation and bioactivity. The characterization for mesenchymal stem cell markers is available elsewhere [19]. Cells were cultured in DMEM (Dulbecco modified Eagle medium (Invitrogen, Carlsbad, CA, USA)) supplemented with 10% fetal bovine serum (Invitrogen) and 1% penicillin/streptomycin (Invitrogen) [46]. Cell were passaged (TrypLE Select, Invitrogen, Carlsbad, CA, USA) at 60–70% of confluence.

To prepare the extracts, the cements were inserted into molds (5 mm in diameter, 2 mm in thickness) and allowed to set for 24 h. The discs were immersed individually in 10 mL of culture medium for seven days. After, the disks were discarded, the eluent filtered (pore size 0.22 µm) and kept at 4 °C for no longer than two weeks. 

For cell viability (*n* = 3), 10 × 10^3^ cells were seeded in 24 well plates with basal medium and left undisturbed for 24 h. Later, cells were treated with wither basal medium (Control) or with extracts for five days. After three and five days, cells were treated with NucBlue^®^ Live and propidium iodide (ReadyProbes^®^ Cell Viability Imaging Kit, Blue/Red, Life Technologies, Carlsbad, CA, USA) for 15 min and imaged under fluorescence microscope DAPI and RFP filters. For bioactivity (*n* = 3), 3 × 10^4^ cells were seeded in 24 well plates and treated with the extracts for 14 days. The mineralization was assessed using Alizarin Red S staining (ARS). Briefly, cells fixed in 4% formaldehyde at room temperature for 30 min. The cells were washed twice with distilled water and 40 mM ARS (pH 4.2) was added to each well. The plates were incubated at room temperature for 30 min, washed four times with distilled water (5 min each). Following, 200 μL of 10% acetic acid (*v*/*v*) was added to each well and incubated at room temperature for 30 min. Cells were scraped from the plate, transferred to a microcentrifuge tube, and vortexed for 30 s. The solution was kept at 85 °C for 10 min and transferred to ice for 5 min. The slurry was then centrifuged and 500 μL of the supernatant transferred to a microcentrifuge tube. Finally, 200 μL of 10% ammonium hydroxide (*v*/*v*) was added to the solution. Absorbance of aliquots (100 μL) of the supernatant were measured using a microplate reader (Infinite M200, Tecan, Frankfurt, Germany) at a wavelength of 405 nm. 

Statistical analyses were performed with two-way analysis of variance (ANOVA) followed by post hoc Tukey test (α = 0.05, SPSS V.17, IBM, New York, NY, USA).

## 5. Conclusions

The hypothesis was partially rejected as the addition of GNS negatively affected the push-out strength of ECZ. Nonetheless, GNS may be an alternative to improve the hardness and shorten setting time of bioactive cements. The presence of GNS does not interfere negatively in the formation of calcium hydroxide, calcium carbonate, and pH release profile that are crucial for the bioactivity role and antibacterial properties of the biocements. The decrease in the push-out resistance observed in ECZ may compromise the clinical performance of the material. Hence, despite the improvements observed, the use of GNS cannot be generalized when effective bonding of bioactive cements is of concern.

## Supplementary Materials

The following are available online at www.mdpi.com/1996-1944/10/6/606/s1.
