# Supplementary material for: Graphene Nanosheets to Improve Physico-Mechanical Properties of Bioactive Calcium Silicate Cements
Source: Materials (Basel). 2017 May 31;10(6):606. doi: 10.3390/ma10060606 (PMC5553423; doi:10.3390/ma10060606)
Supplement: Supplementary file 1 [file materials-10-00606-s001.pdf]

Supplementary Materials: Graphene Nanosheets to Improve Physico-Mechanical Properties of Bioactive Calcium Silicate Cements

Nileshkumar Dubey, Sneha Sundar Rajan, Yuri Dal Bello, Kyung-San Min and Vinicius Rosa

Figure S1 shows the fracture modes observed for the push-out bond strength. The addition of GNS did not favoured the occurrence of a particular pattern.

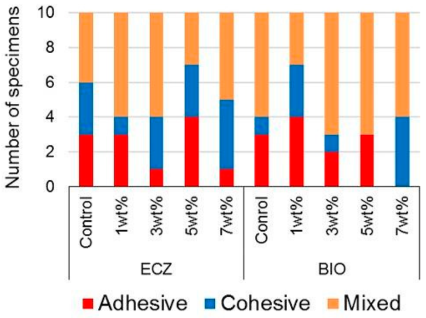

Figure S1. Fracture modes obtained from the push-out bond testing.

Figure S2 shows the dental pulp stem cell viability after 3 (Fig. S2A) and five days (Fig. S2B) under the treatments with extracts obtained. There was no decrease in cell viability for any of the conditions tested for both time points.

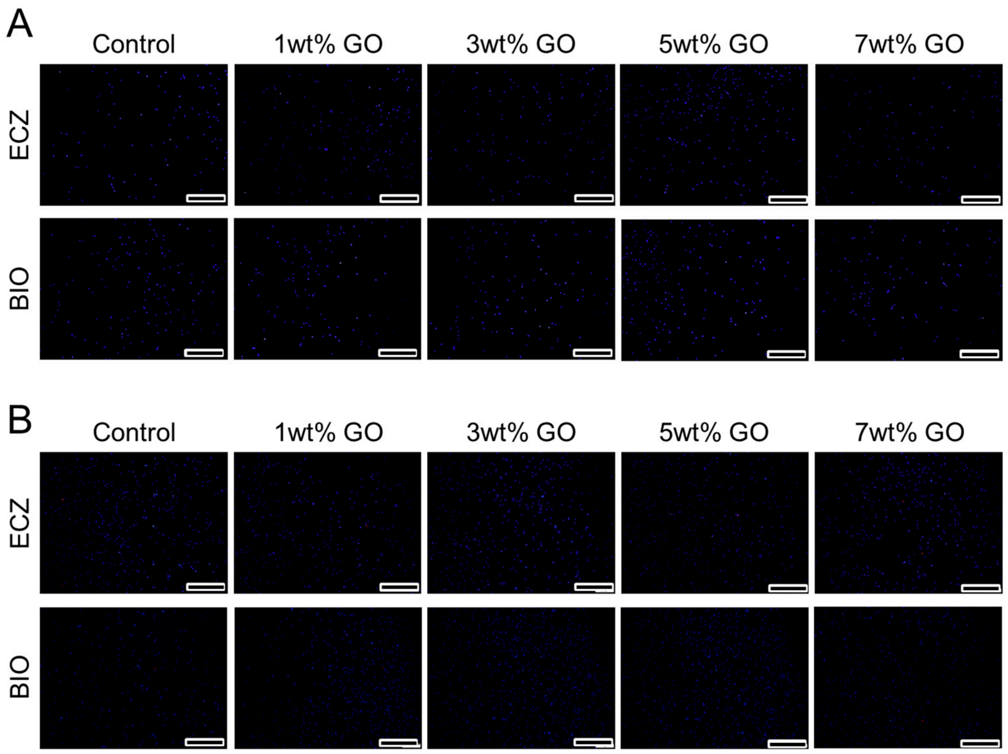

Figure S2. Dental pulp stem cells viability after 3 (**A**) and 5 (**B**) days.
